# Religio, Mores, Cultura

**Published:** 1887-05

**Authors:** A. R. Davidson

**Affiliations:** Prof. of Medical Chemistry and Dermatology


THE BUFFALO Medical AND Surgical Journal

VOL. XXVI. MAY, 1887. No. 10.

### Original Communications.

### "*RELIGIO, MORES, CULTURA.*"

THE DOCTORATE ADDRESS IN THE MEDICAL DEPARTMENT OF NIAGARA UNIVERSITY, 1887. BY A. R. DAVIDSON, M. D., PROF. OF MEDICAL CHEMISTRY AND DERMATOLOGY.

RT. REV. CHANCELLOR, LADIES AND GENTLEMEN:

In the olden time, and to-day in many of the Universities of Europe, while the candidate for the honor of the ordinary scholastic degrees is counted worthy if he fulfill the requirements of the college examination, he who aspires to wear the Hood of the Doctor, either in theology, medicine or law, is compelled to enter the lists with other doctors and in controversial battle prove himself worthy to be admitted to so high an honor and dignity.

Those who have this evening received the high degree of Doctor of Medicine, are victors in a similar contest. The guardian authorities of the University have not only satisfied themselves of their proficiency, but the candidates have passed through the ordeal of a meeting with many eminent physicians, other than their teachers, who have tested their knowledge, and, after a careful examination, have certified that they are worthy of admission to the ranks of the medical profession.

It is only justice to the members of the graduating class to remind the ladies and gentlemen who have honored us by their presence this evening, that there are many doors through which the young man may enter the profession. There are in the United States nearly one hundred and fifty institutions by which the degree of Doctor of Medicine is conferred, and these all fall into one of two classes.

First, the medical college, usually called a university, perhaps because in this growing country there is a possibility that at some future time it may develop into one, or, perchance, because the men connected with it hold themselves in readiness to teach medicine to any one in the universe, without any requirements other than two hundred dollars from each, strictly in advance. Being established on a purely business basis, they offer the degree upon easy terms, say eight or ten months' attendance upon lectures, together with, of course, the usual fees, and twenty-five dollars for graduation.

The more doctors are turned out, the more dollars are turned in—to the professors' pockets; therefore, examinations must be easy and no preliminary education is needed. The necessary dollars and a few months' nominal instruction only, are required to convert the ignoramus into the doctor.

I do not mean to say that the majority of this class of colleges are simply "Diploma Mills," but it is true that in most of them no effort is spared to have as many students and graduates as possible, regardless of quality, and so we see graduating classes of fifty to several hundred.

On the other hand, there are universities with their medical departments, where medicine is taught as one of the learned professions. The candidate for professional honors is met at the very commencement of his course with the question: Have you the education necessary to enable you to intelligently study medicine? and this must be answered either by the production of a scholastic degree or other certificate of proficiency before he can enter the portals of the college, for neither past experience nor present theory warrants the belief that plough-boys, ignorant of the first rudiments of knowledge and untrained in the habits of study, can advantageously pursue the practice of medicine.

Then follows a long course of training in the college, the hospital, the laboratory and at the bedside, extending over at least three years and often four, and terminated by a rigorous examination, not only by his teachers, but also by independent physicians in active practice. He graduates, if he graduates at all, a well-trained and accomplished physician, worthy to wear the Hood of the Doctor of Medicine, and to assume the awful responsibility of the care of human life.

I can imagine this decision in your mind, "That is the kind of Doctor I should wish a son or brother of mine to be," but remember that it takes no little courage for the majority of young men to take the harder and exacting course. Indeed, the requirement of a proper preliminary examination would prevent, at least, half of the present students of medicine from making poor doctors of themselves.

The graduating classes of universities, which in their medical departments uphold a high standard of professional education, will always be small. The tendency will be continually to make fewer doctors and better ones. The graduates of such schools will deserve the honors which will surely come to them.

Gentlemen of the graduating class, I could, for the honor of the profession, wish that your number was larger; but I point to your fewness with pride, as an indication of the faithfulness with which Niagara University has upheld the ideas of reform in medical examination. Of a class of thirteen who entered with you upon the arduous path, four only have attained the honors of a degree from this university.

Gentlemen, your teachers admire the courage, determination, and industry which have animated you during these long years of effort. Most heartily do we salute you Doctors of Medicine, congratulating you upon honors fairly won and which we trust will be worthily worn.

Upon the open book which occupies the centre of the symbolic devices which ornament the university diploma, are the words *Religio, Mores, Cultura.*

Taking these words as my text, I could wish that I had the eloquence and ability of some of the distinguished men before you this evening, that in this hour of greeting and of parting, full of rejoicing over present success and full of hope and glorious promise of new honors in the real work of your life upon which you are now entering, I could make fitting speech—could utter words helpful unto you, whose future perils are many and great, whose opportunities likewise are many and great, and, therefore, whose responsibilities are so grave.

As the soil, however rich it may be, cannot be productive without cultivation, so the mind without culture can never produce good fruit. During the last four years, you have devoted yourselves to the acquirement of medical knowledge. You have laid well the foundations upon which you must continue to build all your lives. Do not forget that to-day, more than at any other previous period of the world's history, medicine is a progressive science. You can keep abreast of your profession only by constant study.

Do not, however, be content with the acquisition of purely medical knowledge. We frequently hear teachers proclaiming that our science is a jealous mistress; that success will come only to those who concentrate their whole time and thoughts upon medicine alone.

Now, I do not believe that the young physician, who, in the first years of his practice, is fortunate if he has upon the average one patient a day, can profitably devote all his unemployed time to the study of medical books. The man who, failing to perceive the relativity of all knowledge, confines himself to any one department, is sure to become narrow-minded and mentally dyspeptic.

But it is impossible that you should have passed through the scientific training essential to your calling without having had more or less enjoyment in fields of knowledge not strictly medical; for, "though not exact and shapely in all her proportions, Medicine may be said to hold princely rank among her sister sciences. She levies tribute from each and all, that it may be melted anew in her crucible, stamped with her image and superscription and distributed for the common good."

In acquiring your education, you have necessarily gained some knowledge of the collateral branches of science. During your student days, the stern necessity of perfecting yourselves within a certain range of knowledge compelled you to turn away from these inviting fields; but now I would urge you to take up some of these studies most congenial to you. These will not only afford you abundant opportunity for healthful diversion, but, with culture and a little technical skill, will enable you to "read in the book of nature lessons of life and death too deep for the conning of the uninstructed swain, while all your finer senses wake to the beautiful and joy with ravishment." It is such culture that makes the man a better physician, the physician a better man, well developed in all directions. *Totus teres atque rotundus.*

Associated with the caduceus, the symbol of medicine, engraved on your diplomas, I see the emblems of music, poetry and art, which, perhaps some would say, were the farthest possibly removed from association with your profession; but you will not forget that the famous Hypocratic oath begins with these words: "I swear by Apollo, the physician, by Æsculapius, by his daughters, Hygeia and Pomaria, and by all the gods and goddesses." The great Bacon says it was well for the poets to conjoin music and medicine in Apollo, because the office of medicine is but to tune this curious harp of man's body and reduce it to harmony; and to the more advanced medical mind of to-day, therapeutics has but a single aim—that to restore harmony in the human organism when disturbed.

We would claim, then, that medicine is not only one of the fine arts, but the finest among them, because it has to do with human life. "See deep enough and you see musically, the heart of nature being everywhere music, if you can only reach it," says the rugged Carlyle. Jonathan Hutchinson, than whom perhaps none of England's famous physicians has gained higher honor, advocates, as a means of intellectual training, a study of he English poets.

A knowledge of drawing, at least to the extent of an ability at free-hand sketching, is within the reach of almost every one; and the ability to make enduring records of cases—to picture and preserve the revelations of the microscope, together with the sharpening of one's power of observation which such practice involves—will be more and more appreciated as you grow in years.

Again, the telescope will remind you of that glorious and mind-expanding science of astronomy; and if we, in the cities, are to some extent shut off, by the petty edifices of man, from viewing the spacious firmament, certainly the physician whose lot is cast in the country will have frequent enough opportunity to beguile his lonely midnight drives by a study of the stars. Here the illiterate man sees only a dome of jewels, resplendent in their flashing beauty, but to the man of books the grand panorama of dazzling beauty and gorgeous display grows in magnitude and splendor. With what a perception of the Divine harmony of things and the majesty of the Creator does he contemplate the dome that encompasses the world. To him it expands into illimitable space, and the jewels which flash with the brilliancy of light grow into worlds and suns and systems, which sweep their mighty curves in obedience to the Divine law.

The retort, the balance and the microscope irresistibly compel me to invite you to pursue, as a most efficient means of mental culture, that science which, dealing with the proportions and affinities of the ultimate parts of matter, is the stem, by plucking which you will come in possession of a whole bunch of fruit. "Chemistry ministers to the esthetic sense in every test, and the microscope opens to view a world of dainty beauties, while both alike suggest to the student conceits for the fancy and themes for exalted thought.

The first admonishes him ever of the reign of Law, and that matter obeys the inflexible mandates of a power impersonal and cold—from the infinitely great to the infinitely small; from world to atom; from the starry systems of space to the molecules of the water-drop. The light, soft, white precipitates and color changes in the test-tube suggests the fleecy skirts of the summer clouds, or the feathery fall of the wintry snow; or bring to mind that trick of Nature's thaumaturgy which decks the trees with green in springtime, the flowers with rainbow hues in summer, and leaf or fruit with gorgeous dyes in the mellow autumn."

You have learned how to use the test-tube and the flask, the burette and the hydrometer, as well as to manipulate your own microscope. Let me urge upon you to add to the knowledge you now have. Every doctor can have a laboratory. Expensive apparatus is not really necessary; remember that Priestly made his magnificent discoveries with apparatus which you would turn from as ridiculously insufficient.

The physician who is familiar with the chemical significance of the morbid variations of the fluids of the body, will be secured against many mistakes. "Instead of groping in the dark and whacking away blindly, sometimes hitting the disease and sometimes the patient—as is the way of some doctors—he will find that his path is illuminated by a flood of light, throwing disease into bold relief, illuminating its path to the end, and showing with distinctness the effects of remedies. Further, he will find a refuge in his laboratory from many of the smaller frets of life, and be cheered by gentle influences unknown to grosser minds. His perception is sharpened by exact study to the better comprehension of the nature of morbid processes, and of the *modus operandi* of medicine. He has a key for solving problems of personal and public hygiene; a light to be got nowhere else for an early detection of some diseases, and sound direction to guide him in their cure."

But, gentlemen, doubtless you have ere this studied out for yourselves the meaning of the emblems which ornament your diploma, and aware that all the arts and sciences are there symbolized; you fear that an allusion to them all is going to lead me into the sin of excessive length and tediousness. But enough! I only ask that, in addition to your medical studies, you seek a broader knowledge. Culture creates a personal independence, and gives a man a larger knowledge and mastery of his own inner self. Cicero tells us that "the cultivation of the mind is the food of the soul."

Not the least of the qualifications for professional success are good manners; but these must be genuine, not sham. The three Graces of manners are Gentleness, Cheerfulness and Urbanity, and these graces you should cultivate, for men succeed less by their talents than their character. Good manners are a part of good morals, and ten men have failed from defects in morals where one has failed from defect in intellect.

Medicine is one of the learned professions; it is also called a liberal profession—that is, its members are required to avoid whatever is mean and low; to shun narrow-mindedness; to be catholic in the discharge of professional duties, irrespective of religion, rank or race; to conduct themselves as gentlemen. The illustrious Thackary thus answers the question, What is it to be a gentleman? "It is to have lofty aims, to lead a pure life, to keep your honor virgin, to have the esteem of your fellow-citizens and the love of your fireside, to bear good fortune meekly, to suffer evil with constancy, and through evil or good report to maintain truth always. Show me the happy man whose life exhibits these qualities, and him we will salute as gentleman, whatever his rank may be." If this is your ideal, I need say nothing of your duties to your fellow-physicians. The gentleman needs no code to regulate his conduct.

But your profession imposes upon you peculiar duties and responsibilities to those who place themselves under your care. "Who stands so close to the needs of man as you will—to whom, in their very direct needs, all will cling– to whose voice sick hearts will listen as if it were the very voice of God declaring judgment or mercy; the very skirts of whose garment, if only a heart beats beneath them, faint hands will be raised to touch; closest to the very sources of life you will stand; it is the physician's place." Endeavor to realize these responsibilities. Let no familiarity with suffering make you cold or unsympathetic, but, humbly following in the footsteps of the

Great Physician, let your heart be a fountain of charity, kindness and love. In the course of your practice, you will learn many personal and family secrets; you may hear many startling revelations. Betray not the confidence reposed in you, or honor and happiness will not be in your path.

And now there remains of my three heads the subject of Religion. It is with hesitation that, in the presence of those who have devoted their whole life to the holy duties of religion, and who have received the Divine commission to teach men the way of salvation, that I address you on this subject. I desire to express my conviction, however, that our culture and manners, and all the good things which are connected with the civilization of to-day, depend upon, and have been evoked by the spirit of religion. There is nothing which so harmonizes with the natural greatness and dignity of human nature as a faith which not only promises the entire refinement of the mind, but also the glorifying of the body. On the other hand, for the medical man to seek a broad culture and the attainment of high moral excellence is doubly necessary, for the undoubted tendency of medical study, when restricted to the mere physical man, is to lead him to the narrowness and absurdity of materialistic theories. Cardinal Newman has wisely said: "A medical philosopher who has so simply fixed his intellect on his own science as to have forgotten the existence of any other, will view man, who is the subject of his contemplations, as a being who has little more to do than to be born, to grow, to eat, to drink, to walk, to re-produce his kind, and to die. He sees him born as other animals are born; he sees life leave him with all those phenomena of annihilation which accompany the death of a brute. He compares his structure, his organs, his functions with those of other animals, and his own range of science leads to the discovery of no facts which are sufficient to convince him that there is any difference of kind between the human animal and the brute."

I sincerely hope that the graduates of Niagara University will never be among those who accept fancies of the most conjectural character as new articles of belief, which involve the abandonment of old truths as well as the sacrifice of the firm bulwarks of faith. The atheistic and materialistic conclusions, so loudly proclaimed by certain scientific men, are accepted by some who are too lazy to think over the principles upon which the doctrines they are persuaded to accept are based; by many, who, impressed with evidences of extraordinary talent in certain fields of science, and accustomed to receive the dicta of scientific men without question, credit them with similar authority on matters of faith, forgetting that the very devotion of mind, through the best years of life, to a totally different class of ideas, render them less instead of more trustworthy upon theological and historical questions.

I believe that all materialistic doctrine, vary as they may in detail, accept as a truth the monstrous assumption that the living and non-living differ only in degree, and that every living thing, every human being, is just as much a machine as a watch, a wind-mill, or a locomotive; that this world of machines is dominated by a blind, relentless, irresistible fate, falsely called law or nature; a nature destitute of intelligence and reason, devoid of justice and mercy, not contributing to the happiness or enjoyment of any, but ever working like a dead, senseless machine of overwhelming might, marring, crushing, distorting, destroying, and thus continuing and preserving.

Those who so authoritatively propound such dogmas appear to look upon themselves as mechanisms of culture and quality, so transcendent that their escaping gases should serve as the motive power of all other machines of humbler capabilities, the threatened penalty of refusal being that of being numbered amongst the fools, the bigots, the orthodox and the like.

But, gentlemen, to the end of the world there will be those who choose darkness. It is light that enables us to see the differences between things, and it is Christ that gives us light. Sir Humphry Davy thus eloquently writes on the advantages of a firm religious belief, which, he says, I would prefer to every other blessing, "For it makes life a discipline of goodness; creates new hopes when all hopes vanish, and throws over the decay, the destruction of existence, the most gorgeous of all lights; awakens life even in death, and from corruption and decay calls up beauty and Divinity; makes an instrument of torture, and of shame a ladder of ascent to Paradise, and, far above all combinations of earthly hopes, calls up the most delightful visions and plains and amaranths, the gardens of the blest—the security of everlasting joys, where the sensualist and the skeptic view only decay, annihilation and despair."

And thus, my new brothers in the profession, I give you farewell counsel. Set out on your career with brave hearts and a firm resolve to do no discredit to your *alma mater*. You bear her name—sustain her reputation by your self-respect, your gentlemanly conduct, your large-mindedness; let no necessity compel, no temptation seduce your feet to devious paths, but with onward stride through every difficulty, danger and trial, may you "Press toward the mark for the prize of your high calling;" and remember that—

> "We live in deeds, not years; in thoughts, not breaths;
> In feeling, not in figures on a dial:
> We should count time by heart-throbs. He most lives
> Who thinks most, feels the noblest, acts the best."

